# Mediators of Atherosclerosis in South Asians Living in America: Use of Web-Based Methods for Follow-Up and Collection of Patient-Reported Outcome Measures

**DOI:** 10.2196/resprot.5448

**Published:** 2016-06-08

**Authors:** Namratha R Kandula, Ankita Puri-Taneja, David E Victorson, Swapna S Dave, Alka M Kanaya, Mark D Huffman

**Affiliations:** ^1^ Feinberg School of Medicine Division of General Internal Medicine, Department of Preventive Medicine Northwestern University Chicago, IL United States; ^2^ Feinberg School of Medicine Northwestern University Chicago, IL United States; ^3^ Feinberg School of Medicine Department of Medical Social Sciences Northwestern University Chicago, IL United States; ^4^ UCSF School of Medicine University of California at San Francisco San Francisco, CA United States; ^5^ Feinberg School of Medicine Department of Preventive Medicine Northwestern University Chicago, IL United States

**Keywords:** cardiovascular diseases, cohort studies, Internet, South Asian

## Abstract

**Background:**

A key challenge for longitudinal cohort studies is follow-up and retention of study participants. Participant follow-up in longitudinal cohort studies is costly and time-consuming for research staff and participants.

**Objective:**

This study determined the feasibility and costs of using Web-based technologies for follow-up and collection of patient-reported outcomes in the Mediators of Atherosclerosis in South Asians Living in America (MASALA) study.

**Methods:**

The MASALA study is a community-based cohort of 906 South Asians in the United States. Since the baseline in-person visits (2010-2013), a yearly telephone follow-up survey was used to assess participants’ health status and incidence of cardiovascular disease. A Web-based version of the follow-up survey was developed using the REDCap (Research Electronic Data Capture) Web app. Participants from the Chicago field center who were due for their annual follow-up and who had a valid email address were sent an email link to a secure online portal where they could complete the survey. Telephone follow-up was used with nonresponders.

**Results:**

A link to the Web survey was emailed to 285 participants (February to October 2014) and the overall completion rate was 47.7% (136/285). One-third of participants who were unresponsive (n=36) to annual telephone follow-up completed the Web survey. Web responders were younger, more likely to be married, and to have higher education and income compared (*P*<.05) to telephone-only responders. Web survey development involved 240 hours of research staff time. Since launching, the Web-based survey has required 3 hours per week of staff time.

**Conclusions:**

Although electronic follow-up will not be a panacea for cohort operations, it will serve as an adjunctive strategy to telephonic follow-up for maximizing cohort retention with lower costs.

## Introduction

Population-based cardiovascular cohort studies contribute important scientific information about risk factors and pathophysiology of cardiovascular disease by collecting high-quality detailed data linked to longitudinal outcomes. However, longitudinal follow-up of cohorts can be costly, time-consuming, and burdensome to research staff and study participants. The balance of cost, data validity, and feasibility is increasingly important for determining the value of traditional population-based cohorts [1]. To remain relevant in a technologically evolving world, cohort studies should be able to capture data using newer methods, such as Web-based follow-up, without sacrificing participant retention rates [2,3]. This study determined the feasibility and costs of using Web-based technologies to longitudinally follow participants in a cardiovascular cohort study, the Mediators of Atherosclerosis in South Asians Living in the United States (MASALA) study.

## Methods

The MASALA study is a community-based longitudinal cohort study designed to understand the risk factors and etiology of cardiovascular disease (CVD) among South Asians living in the United States aged 40 to 84 years who were free of CVD at baseline [4]. Baseline clinical visits were conducted from 2010 to 2013. Once per year, follow-up was conducted using a brief telephone survey to assess changes in participants’ health status, hospitalizations, procedures, and self-reported CVD events. In 2013, the Chicago site received pilot funding to implement and test the feasibility of using Web-based technology for participant follow-up and patient-reported outcomes (PRO) measurement and data collection. Starting in 2014, participants from the Chicago site who had provided a valid email address were given the option of completing the annual follow-up using a Web-based survey. This study was approved by the Institutional Review Board at Northwestern University, Chicago, IL, and informed consent was administered to all participants at the study baseline visit.

### Choice of Web-Based Platform

Initially, the study team evaluated three Web-based platforms: SurveyGizmo, Assessment Center [5], and REDCap (Research Electronic Data Capture) [6]. SurveyGizmo is a proprietary electronic survey tool that was used for follow-up of cohort participants from the Coronary Artery Risk Development in Young Adults (CARDIA) study and is compliant with the Health Insurance Portability and Accountability Act (HIPAA). However, concern about a third-party website hosting study data was an important barrier for using SurveyGizmo. Assessment Center is a platform funded by the National Institutes of Health that was specifically created to collect PROs [7] and to reduce respondent burden. It is widely used by biomedical and behavioral research programs. REDCap is a consortium developed among various institutions for research data capture, including electronic surveys. Both Assessment Center and REDCap platforms are secure, are HIPAA compliant, and allow easy data export. Both Assessment Center and REDCap utilize email as a means of sending the survey link to participants.

REDCap was chosen as the Web-based platform for the MASALA study because it had several necessary features and capabilities that were not available through Assessment Center, such as branching capacity and dynamic templates. Assessment Center can be customized, but the cost of customization was prohibitive for the MASALA study.

### Development of Web-Based Survey

**Figure 1:**
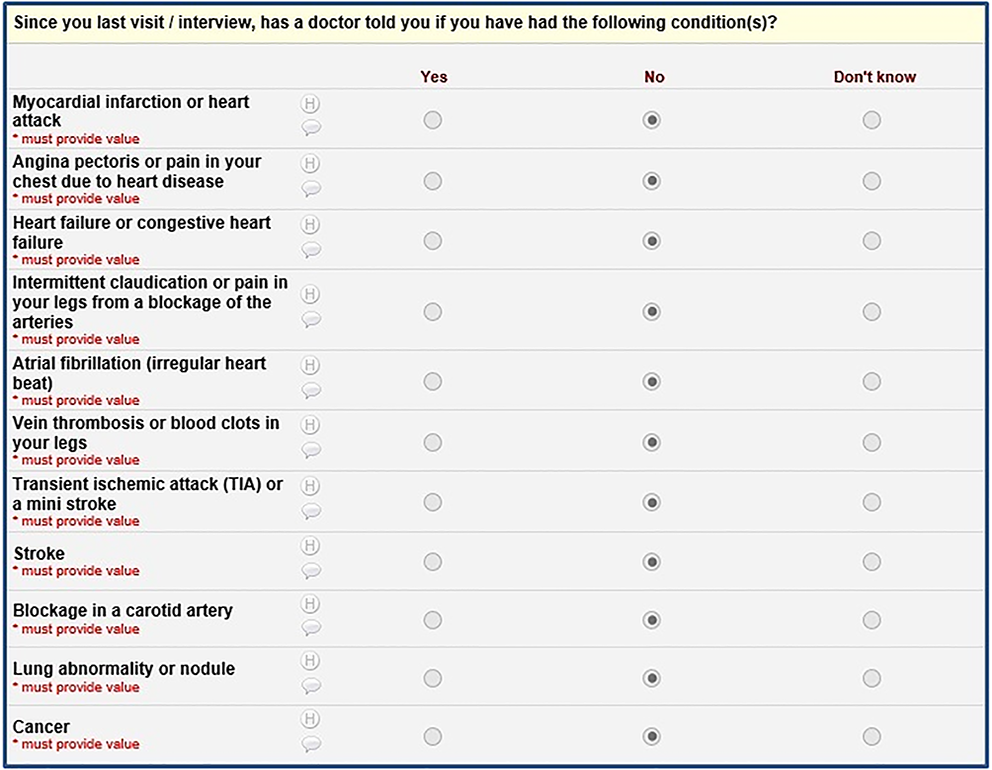
Matrix included in the Web-based questionnaire.

The MASALA annual follow-up survey used paper surveys and the TeleForm version 9.1 software system (Verity Inc, San Francisco, CA, USA) for automated data entry; the survey was converted by study staff into a Web-based survey in REDCap. If a participant reported a hospitalization, CVD event, or other cardiac testing, they were asked to provide further details on the event date, hospital or clinic, and physician’s name so that MASALA staff could obtain the records for event adjudication. In addition to the 21 annual follow-up questions, we added three PRO measures (15 new questions on physical function, applied cognition, and satisfaction with social roles) from the static PRO short forms developed by the Patient-Reported Outcomes Measurement Information System (PROMIS) to the end of the Web-based follow-up survey. These questions were added to collect pilot data for future studies related to aging and cognition among MASALA participants (Figure 1).

We conducted beta testing of the Web-based electronic MASALA (e-MASALA) follow-up survey and PRO using REDCap with 10 South Asian men and women aged 27 to 77 years. Participants varied in their computer literacy and familiarity with the Internet. All participants were able to follow the on-screen instructions, complete the survey, and reported that they understood the questions and response options. When asked about preferences related to how the survey would be viewed in the browser, seven of 10 preferred a one-page survey. Participants also preferred using a matrix to answer questions that had the same stem rather than repeating the stem for each question. The first version of the Web questionnaire required approximately 240 hours of a research assistant’s (RA) time to design, test, and implement.

### Web Survey and Security

Participants received a link to the survey through their email. The survey linked to an online portal from which patients could gain access to a study-specific REDCap page. REDCap allows for secure Web authentication, data logging, and Secure Sockets Layer (SSL) encryption. REDCap is built around HIPAA guidelines, but is not suitable for clinical trials governed by the Federal Drug Administration because it is not compliant with 21 CFR Part 11 [8]. The participant’s study ID and an acrostic were the only identifiers for completed surveys.

### Protocol for Web-Based Survey Follow-Up

Before implementing the Web-based survey, annual follow-up of MASALA participants was completed by telephone using teleforms. If a participant was not reachable after six telephone calls, project staff would mail the teleforms, instructions, and a stamped envelope addressed to the site principal investigator so teleforms could be returned.

The Web-based follow-up survey was sent out to participants who were due for their annual follow-up on the first Friday morning of each month. Four weekly reminders were sent out every Friday if the participant did not complete the survey in the prior week. The participant’s baseline clinical visit date determined when the annual follow-up survey was sent. During the initial 2 months of this pilot study, the Web-based survey was also sent to participants who had been unresponsive to telephone follow-up since their baseline visit. Participants who did not complete the Web-based survey after four reminders were contacted at the end of the month by telephone to complete their annual follow-up. If time allowed, the RA asked participants about reasons for not completing the Web-based survey.

Initially the participants received three emails over a 4-week period to complete the survey. After observing the first month’s completion rate, a fourth and final reminder email was added. This fourth email resulted additional survey completions without any obvious increase in participant burden. We continued to send four emails and the final email for the month was sent with the subject heading: “Final reminder: Last chance to complete your annual MASALA follow-up survey via Internet.” This subject heading used a deadline and time-sensitive language to create a sense of urgency in participants [9].

The participants were emailed on Friday each week. The decision to send the email link to the survey on a Friday was based on study staffs’ prior experience contacting MASALA study participants. Previously, we found that participants were more likely to read study recruitment letters or respond to study phone calls on Fridays and over the weekend because they had more time. Thus, we used the same protocol for the Web survey.

RedCAP is equipped with a scheduling feature that can be used to schedule and automate scheduled email reminders on a particular day and time. This feature was important for reducing staff burden during holiday season and during long weekends while ensuring that participants received their emails reminders weekly.

Descriptive statistics were used to calculate the completion rate for the Web survey to compare Web responders to telephone responders and to calculate estimated costs of using a Web-based survey for follow-up data collection. Differences between Web responders and telephone responders were compared using unadjusted chi-square test and *t* test for age with a *P* value of .05 or less to determine statistical significance. Analyses were conducted using SAS version 9.4 software (SAS Institute, Inc, Cary, NC, USA).

## Results

To date, we have completed 9 months of data collection using the Web-based platform at the Chicago field center of the MASALA study. A link to the follow-up survey has been emailed to 285 participants and the overall rate of completion was 47.7% (136/285) (Table 1). The majority of surveys were completed on the same day that the first email was sent. There were demographic differences in participants who completed the follow-up survey by Web or telephone. For example, Web responders were significantly younger and more likely to have higher education and incomes than telephone-only responders (Table 2). Two participants reported a CVD event using the Web-based survey.

**Table 1:** Completion rates of a Web-based follow-up survey in the Mediators of Atherosclerosis in South Asians Living in America (MASALA) study, Chicago field center, 2014 (N=285).

Month (2014)	Total surveys emailed, n	Weekly survey completion, n				Completed Web-based survey, n (%)
		Week 1	Week 2	Week 3	Week 4	
February	66	5	9	3	—^a^	20^b^(33)
March	58	10	6	9	13	39 (67)
April	20	5	6	2	0	11 (55)
May	22	3	2	1	0	6 (27)
June	28	7	3	6	1	17 (60)
July	18	5	2	2	0	9 (50)
August	25	2	3	3	3	11 (44)
September	29	1	5	5	4	15 (52)
October	19	2	3	2	1	8 (42)

^a^Fourth email reminder added in March.

^b^Three follow-up surveys were completed by telephone when participants called us in response to e-MASALA.

**Table 2:** Characteristics of MASALA study participants in the electronic pilot study by response modality, 2014 (N=285).

Characteristics		Web survey (n=119)	Telephone (n=125)	No response (n=51)	*P*
Sex (female), n (%)		50 (42.0)	61 (48.8)	20 (39)	.41
**Marital status,**^a^ **n (%)**					.02
	Married	116 (97.4)	110 (88.0)	48 (94)	
	Unmarried	3 (2.5)	15 (12.0)	3 (6)	
Age (years), mean (SD)		55.4 (9.5)	57.5 (9.0)	51.9 (9.0)	.002
**Age group (years), n (%)**					.02
	35-44	18 (15.1)	13 (10.4)	15 (29)
	45-54	43 (36.1)	34 (27.2)	19 (37)	
	55-64	35 (29.4)	49 (39.2)	10 (20)	
	65-74	19 (15.9)	26 (20.8)	5 (10)	
	75-84	4 (3.3)	3 (2.4)	2 (4)	
**Education, n (%)**					<.001	
	≤ High school	2 (1.6)	19(15.2)	4 (8)	
	Some college/bachelors	35 (29.4)	54 (43.2)	21 (41)	
	≥ Bachelors	82 (68.9)	52 (41.6)	26 (51)	
**Income group (US$), n (%)**					<.001	
	<50,000	16 (13.4)	43 (36.4)	13 (25)
	50,000-100,000	24 (20.1)	27 (22.8)	9 (18)	
	100,000-200,000	36 (30.2)	35 (29.6)	15 (29)		
	>200,000	41 (34.4)	13 (11.0)	14 (27)	
					

^a^Marital status of 21 nonrespondents is not known.

One-third (12/36) of participants who were unresponsive to telephone follow-up since their baseline exam (n=36) completed the Web-based survey. Some surveys were partially completed (n=8), where participants opened the link but did not complete the survey. A personal reminder from the site principal investigator was sent to participants who opened, but did not complete, the survey. After receiving the reminder email, six of eight participants revisited and completed the Web-based survey.

Among participants who were contacted by telephone to complete the follow-up survey and who were asked about reasons for not completing the Web survey (n=32), most (78%, 25/28) said it was because they were too busy, did not check their email regularly, or did not pay attention to the email. These participants said that they would try to complete the Web-based survey in the future because they were subsequently aware of it.

An RA developed and implemented the Web survey. During the initial development stages, the RA spent 20 hours per week for 8 weeks exploring different platforms and their capabilities (Table 3). This time was primarily spent on exploring different Web platforms and designing and implementing the Web-based survey. During survey development in REDCap, the RA spent 10 hours per week meeting with REDCap staff, designing the branching and skip patterns, and pretesting and modifying the Web survey.

**Table 3:** Comparison of approximate costs for telephone, Web-based, and mixed mode (50% Web-based and 50% telephone) follow-up.

Activity	Survey type (US$)		
	Telephone	Web-based	Mixed method
Development (exploration, instrument building, usability testing)	$8500 (5 teleform pages at $1700/page)	$3600 (at $15/hr)=$2400 (20 hr/wk for 2 months) + $1200 (10 hr/wk for 1 month)	$12,100/year
Operation and maintenance	$3120/year (4 hr/wk at $15/hr)	$2340/year (3 hr/wk at $15/hr)	$3120/year
Data management	$2340/year (at $45/wk)	Included in maintenance	$1170/year
Total annual cost	Year 1: $13,960; Year 2 onward: $5460	Year 1: $5940; Year 2 onward: $2340	Year 1: $16,480; Year 2 onward: $4290

Since its launch, the Web survey and platform required 3 hours per week of the research staff’s time to build new batches of participants who are due for follow-up, send out the surveys, track survey completion, and send follow-up emails to participants who did not complete the survey in the prior week. The process of collecting annual follow-up information via telephone required research staff to make multiple attempts to contact participants, spend 5 to 10 minutes interviewing participants, and then fax the teleforms to the study’s data management center. The annual cost of the telephone follow-up and data management of teleforms was US $13,960 annually (Table 3). During its first year of design and implementation, the annual cost of the Web survey was US $6660 and the projected cost of operating and maintaining the Web-based survey is expected to be US $2340 per year. REDCap provides automated export procedures for data, whereas the telephone survey required manual entry of data onto a teleform and data verification after the teleform was faxed to the data-coordinating center.

## Discussion

This study found that a Web-based platform was feasible and at a lower cost than telephone follow-up for the collection of longitudinal follow-up data in the MASALA study cohort. Of participants who received a link to the Web survey, we found that 48% completed it. Differences between Web responders and telephone-only responders included age, sex, income, and education. Telephone follow-up of participants who did not respond to Web surveys was still required; however, the Web survey allowed research staff to spend far less time on follow-up. Web-based follow-up also lowered the number of telephone contacts between participants and study staff, which may in turn help to reduce participant burden. One important finding was that one-third of respondents who had not previously responded to the follow-up survey completed the Web survey, which suggests that a Web platform can help engage participants who may be difficult to reach by telephone. The majority of costs that were associated with development of a Web survey were for development, beta testing, and initial implementation. Since the initial implementation, the cost of maintaining the Web survey and follow-up data have been minimal compared to the costs of telephone follow-up and management of teleform data.

We had a higher completion rate of the Web survey compared to what was reported in the Black Women’s Health Study; in 2007-2008, the investigators reported that approximately 25% of participants completed the Web survey [10]. However, they noted that completion of Web surveys among participants was increasing over time, suggesting that temporal trends in Internet use would lead to more individuals having access to the Internet and feeling more comfortable with Web surveys. The finding that age and socioeconomic status influenced mode of response has been reported by others [10-14].

In October 2014, the National Heart, Lung, and Blood Council (NHLBI) Board of External Experts Working Group’s Recommendations to the NHLBI Advisory Council stated that, “NHLBI should actively engage in studies to establish the validity, reliability, and scalability of electronic tools for primary data collection” [3]. The board also recommended active support by NHLBI for the development, validation, and sharing of digital tools. Along with the Health eHeart [15] and the CARDIA cohort’s recent experiences, these data represent some of the first describing the methods and findings from “e-epidemiology” in the United States. The primary difference between e-MASALA and studies such as Health eHeart is the lack of an in-person examination among the latter’s participants. Other cohort studies, such as the Nurses’ Health Studies [16] and others, have not included in-person examinations and have relied solely on telephone and postal mail to capture data. Such cohort studies can be efficient, but can also lack in-depth phenotyping with in-person examinations or advanced imaging.

It also remains uncertain how participant retention rates may differ, if at all, among participants who are recruited and followed in an Internet-based cohort compared to a traditional cohort study. A study of the association between communication strategies used for recruitment (offline, online, face-to-face) and follow-up participation in nine Internet-based cohorts found that follow-up participation ranged from 43% to 89% depending on the cohort. The study also found that participants who became aware of the study through an online communication campaign, compared with those through traditional offline media, seemed to have a lower follow-up participation in eight of nine cohorts [17].

Studies have also found associations between sociodemographics and participation in follow-up reporting. In the Influenzanet study, participants from seven European countries were asked to report weekly symptoms during influenza season using a Web-based reporting system [18]. Sociodemographic factors associated with lower participation in follow-up reporting included younger age, lower education, living in a household with children, and not being vaccinated for influenza. However, another Web-based cohort study found that individuals with lower self-reported computer skills and literacy were more positive toward the study and less concerned about the burden of study follow-up than those with higher education [14]. Given this information, it appears that any well-designed cohort study, regardless of how data are collected, should use a combination of Internet and non-Internet engagement and retention activities to enhance follow-up of all participants and to potentially reduce selection bias.

Strengths of this study include novel development, implementation, and evaluation of a Web-based survey instrument within a traditional cohort and inclusion of start-up and maintenance time and cost estimates to help researchers leading other longitudinal cohort studies. However, our study also has limitations. First, we piloted the Web-based follow-up study only among MASALA participants from the Chicago field center. However, we might expect even higher response to Web-based follow-up among the San Francisco-based participants because those participants tend to have higher education and income levels, which were associated with higher use of the Web-based follow-up instrument. This program was expanded to the San Francisco field center in January 2015. Second, our results are limited to South Asians in the United States and results from other race/ethnic groups and nationalities may be different, particularly when differences in sex, education, and income are present. Our ability to demonstrate feasibility and lower costs with Web-based follow-up serve as an initial step toward more advanced methods of data capture.

We successfully implemented a Web-based survey for follow-up and collection of PRO measures among MASALA cohort participants. These results demonstrate the benefits of using Web-based methods for longitudinal follow-up in epidemiologic cohort studies and that a combination of modalities may be most effective. Although electronic follow-up will not be a panacea for cohort operations as hypothesized by some, it will serve as an adjunctive strategy to telephone follow-up for maximizing cohort retention, lower costs, and possibly lower participant and research staff burden. Other traditional cohort studies can adapt these methods for Web-based follow-up of research participants.

## Abbreviations

CARDIA: Coronary Artery Risk Development in Young Adults Study
CVD: cardiovascular disease
e-MASALA: electronic MASALA
MASALA: Mediators of Atherosclerosis in South Asians Living in America Study
PRO: patient-reported outcomes
PROMIS: Patient-Reported Outcomes Measurement Information System
RA: research assistant
REDCap: Research Electronic Data Capture
SSL: Secure Sockets Layer
